# Analysis of 3-Dimensional Arch Anatomy, Vascular Flow, and Postnatal Outcome in Cases of Suspected Coarctation of the Aorta Using Fetal Cardiac Magnetic Resonance Imaging

**DOI:** 10.1161/CIRCIMAGING.121.012411

**Published:** 2021-06-30

**Authors:** David F.A. Lloyd, Milou P.M. van Poppel, Kuberan Pushparajah, Trisha V. Vigneswaran, Vita Zidere, Johannes Steinweg, Joshua F.P. van Amerom, Thomas A. Roberts, Alexander Schulz, Marietta Charakida, Owen Miller, Gurleen Sharland, Mary Rutherford, Joseph V. Hajnal, John M. Simpson, Reza Razavi

**Affiliations:** 1 School of Imaging Sciences and Biomedical Engineering, King’s College London, United Kingdom (D.F.A.L., M.P.M.v.P., K.P., J.S., J.F.P.v.A., T.R., A.S., M.R., J.H., R.R.).; 2 Department of Congenital Heart Disease, Evelina London Children’s Hospital, United Kingdom (D.F.A.L., K.P., T.V.V., V.Z., M.C., O.M., G.S., J.M.S., R.R.).

**Keywords:** biomarkers, heart defects, congenital, magnetic resonance imaging, pediatrics, prognosis

## Abstract

Identifying fetuses at risk of severe neonatal coarctation of the aorta (CoA) can be lifesaving but is notoriously challenging in clinical practice with a high rate of false positives. Novel fetal 3-dimensional and phase-contrast magnetic resonance imaging (MRI) offers an unprecedented means of assessing the human fetal cardiovascular system before birth. We performed detailed MRI assessment of fetal vascular morphology and flows in a cohort of fetuses with suspected CoA, correlated with the need for postnatal intervention.

**Methods:**

Women carrying a fetus with suspected CoA on echocardiography were referred for MRI assessment between 26 and 36 weeks of gestation, including high-resolution motion-corrected 3-dimensional volumes of the fetal heart and phase-contrast flow sequences gated with metric optimized gating. The relationship between aortic geometry and vascular flows was then analyzed and compared with postnatal outcome.

**Results:**

Seventy-two patients (51 with suspected fetal CoA and 21 healthy controls) underwent fetal MRI with motion-corrected 3-dimensional vascular reconstructions. Vascular flow measurements from phase-contrast sequences were available in 53 patients. In the CoA group, 25 of 51 (49%) required surgical repair of coarctation after birth; the remaining 26 of 51 (51%) were discharged without neonatal intervention. Reduced blood flow in the fetal ascending aorta and at the aortic isthmus was associated with increasing angulation (*P*=0.005) and proximal displacement (*P*=0.006) of the isthmus and was seen in both true positive and false positive cases. A multivariate logistic regression model including aortic flow and isthmal displacement explained 78% of the variation in outcome and correctly predicted the need for intervention in 93% of cases.

**Conclusions:**

Reduced blood flow though the left heart is associated with important configurational changes at the aortic isthmus in fetal life, predisposing to CoA when the arterial duct closes after birth. Novel fetal MRI techniques may have a role in both understanding and accurately predicting severe neonatal CoA.

## Abstract 2

Supplemental Digital Content is available in the text.

**CLINICAL PERSPECTIVE**

Predicting critical neonatal coarctation of the aorta before birth is notoriously challenging with a high false positive rate using fetal echocardiography alone. Recent advances in prenatal cardiac magnetic resonance imaging have allowed for the generation of reliable, high-resolution 3-dimensional imaging of the fetal vasculature, as well as the ability to measure vascular flows using retrospectively gated phase-contrast sequences. This study applied these novel magnetic resonance imaging techniques to a cohort of 51 fetuses with suspected coarctation in the early third trimester of pregnancy, of whom 25 of 51 (49%) went on to require early postnatal surgery for critical coarctation. Reduced flow through the left heart was seen in both the true positive and false positive cases when compared with healthy controls and was associated with significant changes in the angulation of the proximal displacement of the aortic isthmus in both groups. A multivariate logistic regression model incorporating ascending aortic blood flow and proximal displacement of the aortic isthmus was highly predictive of the need for postnatal intervention, correctly classifying 95% of the true positive and 91% of false positive cases. Fetal cardiac magnetic resonance imaging may have an important complementary role to fetal echocardiography in predicting critical neonatal coarctation.

**See Editorial by Hornberger and Eckersley**

Coarctation of the aorta (CoA) is one of the most common pediatric cardiac conditions, accounting for around 7% of all live births with congenital heart disease.^1^ In critical, or duct-dependent, CoA, prompt identification and treatment is critical to avoid long-term morbidity or death of the affected neonate. As such, CoA is one of the congenital cardiac malformations with the strongest evidence for improved outcomes following prenatal diagnosis.^2–5^ Accurately predicting which fetuses will go on to develop postnatal coarctation, however, can be extremely challenging, with false positive rates ranging from 20% to 30% up to 80% in some studies.^6–9^ This is likely due, in part, at least, to a cautious approach from clinicians who are understandably concerned not to overlook infants who might prove to have coarctation after birth. Postnatal management for these patients often involves serial echocardiograms as the arterial duct (DA) constricts to confirm or refute the diagnosis, generating significant parental anxiety, as well as being resource intensive for neonatal and pediatric cardiology services.^10^

The exact pathophysiological mechanisms that lead to the development of CoA are still poorly understood, despite the first such theories being proposed over 180 years ago.^11–17^ The so-called branching theory, proposed by Hutchins^14^ in 1971, postulates that reduced or reversed flow at the isthmus leads to the distal arch becoming a functional branch of the DA (Figure 1). This theory is based on the observation, from surgically resected specimens, that the posterior shelf—the ridge of infolded tissue associated with isthmal obstruction following DA closure—is histologically identical to the arterial infolding seen in other major aortic branches. The increased distance between the left subclavian and the left common carotid arteries, often observed in coarctation, is similarly explained by retrograde filling of the left subclavian from the DA.^14^

**Figure 1.**
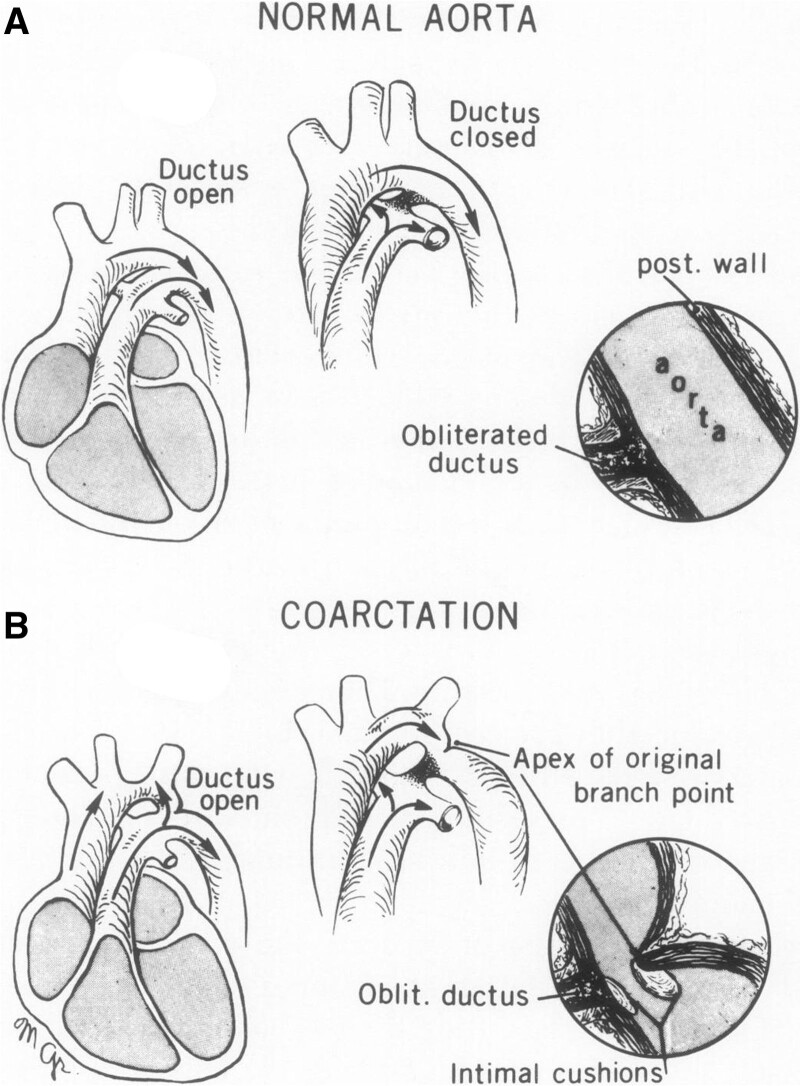
**Coarctation of the aorta explained as a branch-point of the ductus arteriosus. A**, Prenatal and postnatal distribution of normal aortic, pulmonary and ductal blood flow. **B**, Potential mechanism of neonatal coarctation proposed by Hutchins et al. Reduced or reversed flow at the isthmus leads to the distal aortic arch becoming a functional branch of the arterial duct. The apex of this “branch point” is then responsible for the posterior infolding observed in neonatal coarctation, following physiological obliteration (Oblit.) of the arterial duct. Reproduced from Hutchins^14^ with permission. Copyright © 1971.

Recent advances in prenatal cardiac magnetic resonance imaging (MRI) have allowed for the generation of reliable, high-resolution, 3-dimensional (3D) imaging of the fetal vasculature in the early third trimester.^18^ In addition, separate phase-contrast techniques have been developed and validated to measure flows in the major fetal vessels using novel post hoc cardiac gating algorithms.^19^ The aim of this study was to combine these 2 novel fetal MRI techniques to investigate fetuses with suspected CoA, hypothesizing that (1) there is a definable relationship between aortic arch geometry and underlying vascular flow and (2) this relationship can be used to more accurately predict critical or duct-dependent neonatal CoA from prenatal imaging.

## Methods

Patients were recruited prospectively between July 2015 and August 2018 via iFind (Wellcome Trust Innovative Engineering for Health Award 102431)—a large research project focused on the development of novel prenatal diagnostic imaging methods. Ethical approval was obtained (Research Ethics Committee [REC] number: 14/LO/1806), and all subjects gave informed consent. Inclusion criteria were suspected CoA following fetal echocardiographic assessment, with concordant cardiac connections and anticipated biventricular circulation at the time of diagnosis. Cases were identified and offered fetal cardiac MRI at the discretion of the attending fetal cardiologist. Maternal exclusion criteria included weight over 125 kg, severe claustrophobia, inability to give informed consent, or <18 years of age at the time of referral. The data that support the findings of this study are available from the corresponding author upon reasonable request.

Anatomic MRI evaluation consisted of up to 12 overlapping 2-dimensional single-shot fast spin echo sequences (Philips, 1.5T, repetition time [TR], 20 000 ms; echo time [TE], 80 ms; flip angle, 90 °; voxel size, 1.25×1.25 mm; slice thickness, 2.5 mm; sensitivity encoding [SENSE] factor, 2; partial Fourier factor, 0.547; slice duration, 546 ms), processed using 3D motion-corrected slice-volume registration techniques developed and previously validated in our institution, which generate detailed 3D super-resolution volumes of the fetal thorax.^18^ The minimum isotopic output resolution was 1.25 mm interpolated to 0.75 mm for all vascular measurements. No sedation, external gating, or intravenous contrast was used. Fetal vascular flows were measured using 2-dimensional phase-contrast sequence (Philips, 1.5T; TR, 6.78 ms; TE, 3.15 ms; matrix size, 240×240; field of view, 240 mm; slice thickness, 3 mm; simulated R-R interval, 545 ms) planned from orthogonal balanced turbo field echo (BTFE) localizers of the fetal thorax. Individual sequence planes were planned perpendicular to the ascending aorta (AAo), the main pulmonary artery, the superior vena cava (SVC), the DA (duct), the descending aorta (DAo), and the umbilical vein, measured in the fetal abdomen just beyond the insertion point at the umbilicus. All phase-contrast sequences were then retrospectively gated using a technique known as metric optimized gating, according to previous described methods.^19^ The estimated fetal weight was calculated from total fetal body volume, calculated from a single stack of 2-dimensional MRI images with whole-uterus coverage, and converted to fetal weight using an established conversion formula.^20^

**Figure 2.**
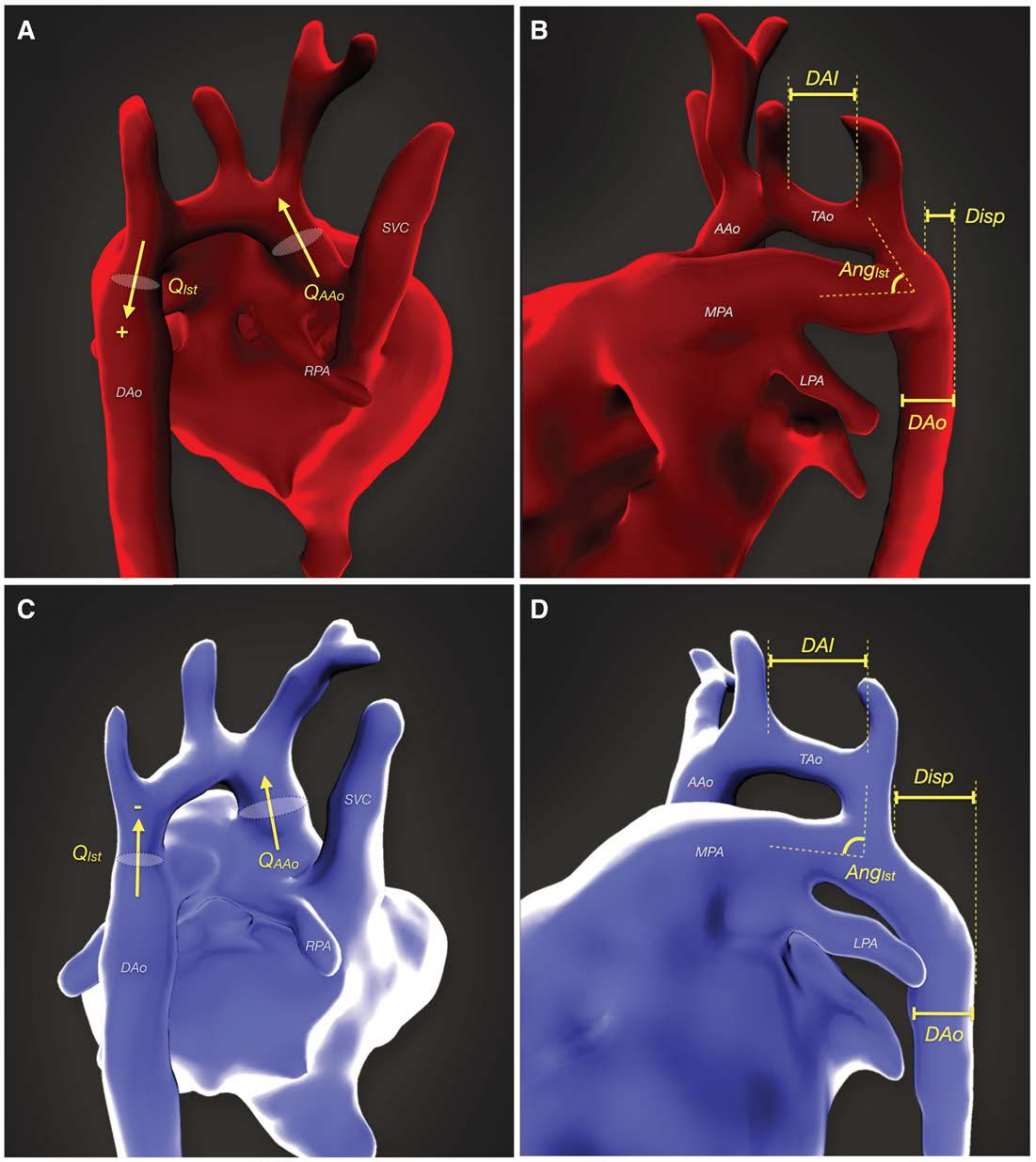
**Three-dimensional fetal cardiac magnetic resonance imaging data from a false positive (red) and true positive (blue) case of coarctation of the aorta, both acquired at 32 wk.** Panels **A** and **C** show the fetal heart from a rightward/posterior position and panels **B** and **D** from a leftward/anterior position. A schematic overlay demonstrates the major measurements undertaken this study. AAo indicates ascending aorta; AngIst, isthmus:ductal angle; DAI, distal arch index; DAo, descending aorta; Disp, isthmal displacement; LA, left artery; MPA, main pulmonary artery; Q_AAo_, ascending aortic flow (mL/kg per min); Q_Ist_, isthmus flow (mL/kg per min; shown as anterograde [+] in **A** and retrograde [−] in **C**); RA, right artery; SVC, superior vena cava; and TAo, distal transverse aortic arch.

Six major parameters were analyzed (2 flow related and 4 anatomic), based on the hypotheses described above (see also Figure 2):

1. The indexed ascending aortic flow (Q_AAo_) measured by phase-contrast MRI with metric optimized gating^19^
2. The indexed flow at the aortic isthmus (Q_Ist_) calculated by subtracting the SVC flow from the AAo flow^19^
3. The angle subtended by the aortic isthmus with respect to the DA (Ang_Ist_)
4. The distance between the posterior wall of the aortic isthmus and the posterior wall of the DAo (isthmal displacement), indexed to the size of the DAo (Disp:DAo)
5. The distance between the left common carotid artery and the left subclavian artery divided by the mean cross-sectional diameter of the aortic arch (the distal arch index, DAI)^21^
6. The ratio of the mean cross-sectional diameter of the aortic isthmus to that of the DA (Ist:Duct ratio).

The angle between AAo and DAo (Ang_AAo-DAo_) and the angle between the transverse aortic arch and the DAo (Ang_TAo-DAo_) were also included, as both were reported as being highly associated with postnatal coarctation in a single retrospective echocardiographic study.^22^ Two observers (D.F.A.L., attending fetal cardiologist with 9 years of general and 5 years of fetal cardiac MRI experience, and M.P.M.v.P., clinical research fellow with 4 years of fetal cardiac MRI experience) performed the entire set of morphological measurements once independently on 3D motion-corrected volumes. The isthmus:ductal ratio, Disp:DAo ratio, and Ang_Ist_ were measured on all subjects on 2 separate occasions, 6 months apart, by D.F.A.L. who also performed all flow measurements. All data were fully anonymized, and both observers were blinded to postnatal outcome at the time of performing measurements. Inter- and intraobserver variability were calculated using the intraclass correlation coefficient (ICC). Unless specified, the mean measurements from both observers were used for further analysis. The degree of correlation between variables was assessed using the Spearman correlation coefficient.

The need for early surgical intervention was determined after birth by echocardiographic assessment according to our previously published approach.^21^ If there was no definitive CoA on initial echocardiography, the aortic arch was observed until ductal closure. All suspected CoA cases were then divided into 2 groups based on whether they required early surgical repair of CoA (<28 days postnatal age; true positive) or were discharged from hospital without intervention (false positive). Mean values for each variable were compared between (1) the healthy control group and false positive group and (2) the false positive and true positive group. Q:Q plots were used to confirm a normal distribution of values, and differences between groups were compared using the Student *t* test. Univariate logistic regression analysis was performed on each variable according to the binary outcome of coarctation repair (yes/no) as per the definition above. After testing for multicollinearity, significant variables were included in a forward-selection multivariate logistic regression analysis. All statistics were performed using SPSS (released 2016, IBM SPSS Statistics for Windows, version 24.0; IBM Corp, Armonk, NY).

## Results

During the time period of this study, 116 patients fulfilling the inclusion criteria were seen in our fetal cardiology department, of whom 55 were referred for MRI assessment (47%). A further 21 healthy controls were recruited, leading to a total of 76 participants in this study. Four patients were unable to proceed with MRI: 1 due to maternal claustrophobia, 1 due to recent fitting of metallic braces, and 2 who did not attend and were unable to be rebooked, leaving a total of 51 patients in the suspected CoA group. The mean gestational age at MRI was 31 weeks (range, 26–38 weeks). 3D reconstructions for morphological measurements were obtained in all 72 patients. Phase-contrast flow measurements were attempted in 61 cases (all 51 CoA cases and 10 healthy control fetuses) and were obtained in 53 patients (87%).

All fetuses were live born. Twenty-five of 51 neonates from the suspected CoA group (49%) were accepted for surgical coarctation repair within the first 28 days of life. Of this group, 11 of 25 (44%) were challenged with temporary withdrawal of intravenous prostaglandin to allow the DA to constrict to confirm the diagnosis before intervention. In the remaining 14 cases, the initial neonatal echocardiographic findings were felt to be clearly diagnostic of coarctation, and PGE infusion was continued to surgery. CoA was subsequently confirmed at surgery in all 25 cases. In the false positive group, 8 of 26 (31%) patients received intravenous prostaglandin initially, which was subsequently withdrawn to permit ductal closure. No patients in this study underwent catheter intervention as primary treatment, and no patients required a concomitant aortic valve intervention. All surgeries were performed during the first admission and before complete closure of the DA. In one case, a plan for surgery was agreed within the first week of life but delayed until 75 days postnatal age due to premature delivery at 30 weeks. For the purpose of further analysis, this patient was, therefore, placed in the confirmed CoA group. The remaining 26 patients (51%) were discharged without intervention, undergoing outpatient follow-up only (false positive). To date, 1 patient in this group has gone on to require late coarctation repair (at 88 days of age). A full summary of all individual patient characteristics is shown in Table I in the Data Supplement.

**Figure 3.**
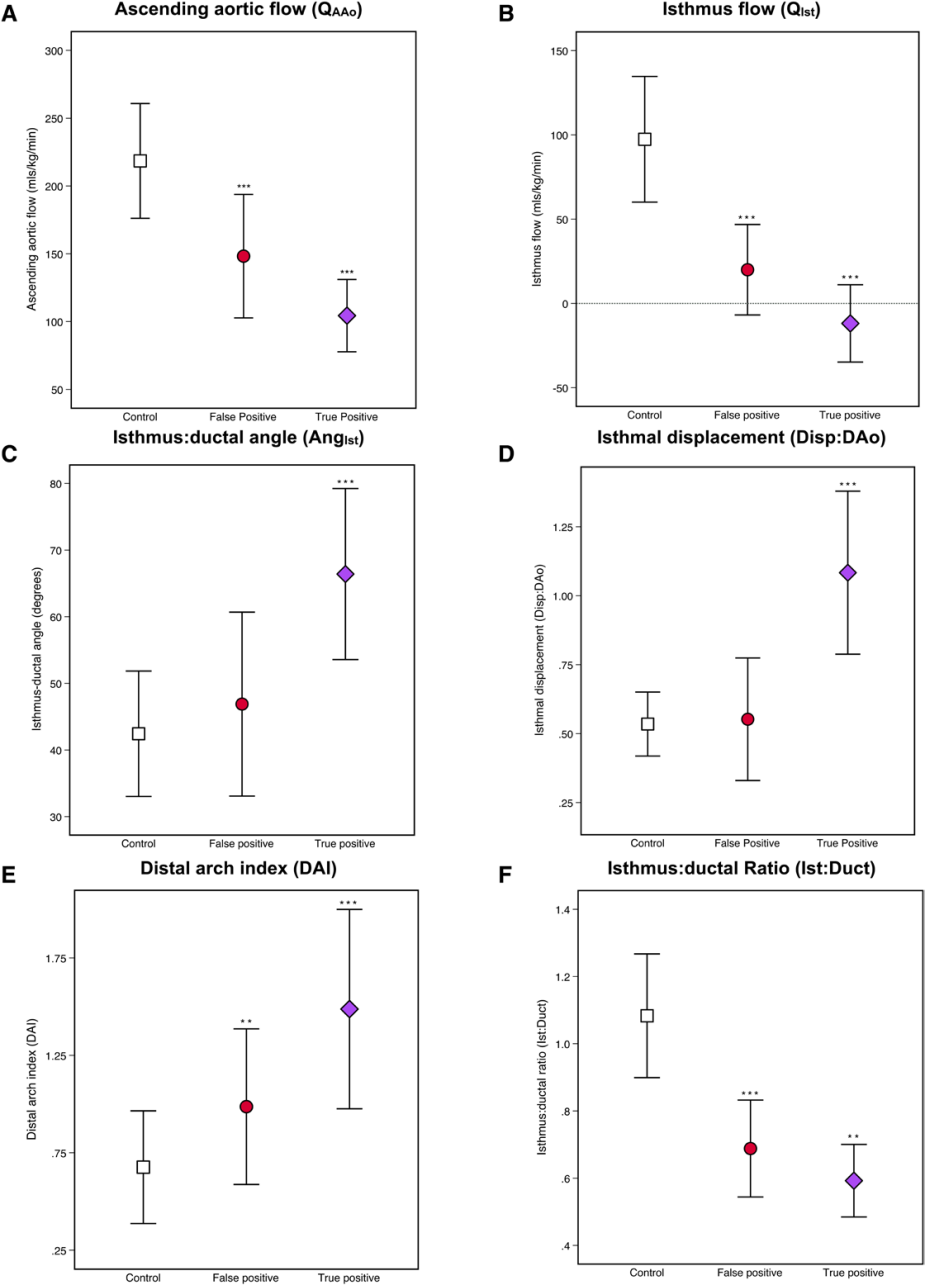
**Error bars showing the mean (±SD) of all phase-contrast flow and morphometric parameters, grouped according to postnatal outcome (confirmed coarctation of the aorta requiring surgery at <28 d postnatal age). A**, Ascending aortic flow (mL/kg per min); **B**, isthmus flow (mL/kg per min); **C**, isthmus ductal angle (degrees); **D**, ratio of isthmal displacement to descending aortic diameter; **E**, distal arch index (distance between left common carotid and left subclavian arteries divided by transverse aortic arch diameter); F, ratio of the diameter of aortic isthmus to the arterial duct. *P≤0.05; **P≤0.01; ***P≤0.001 (calculated with respect to prior category).

**Figure 4.**
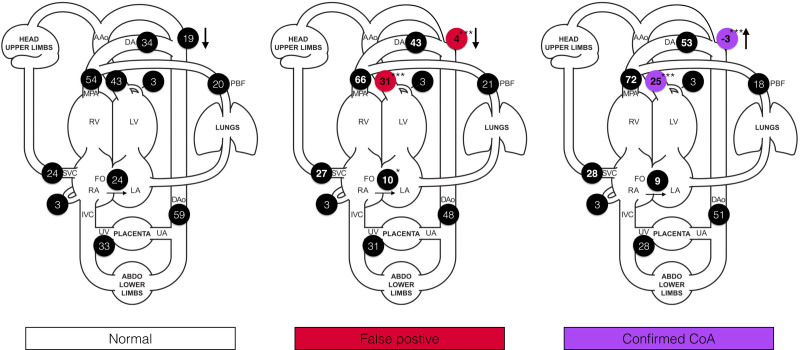
**Topological representation of the distribution of the fetal circulation across the 3 groups studied.** The values displayed represent mean percentage of the combined ventricular output (CVO; 100%). Three percent of the CVO is assumed to represent the coronary circulation (not measured).^38^ Healthy controls are shown in the **left**, false positive cases in the **middle**, and those with confirmed coarctation of the aorta requiring neonatal intervention in the **right**. AAo indicates ascending aorta; CoA, coarctation of the aorta; DA, ductus arteriosus/arterial duct; DAo, descending aorta; FO, foramen ovale; IVC, inferior vena cava; LA, left atrium; LV, left ventricle; MPA, main pulmonary artery; PBF, pulmonary blood flow; RA, right atrium; RV, right ventricle; SVC, superior vena cava; UA, umbilical artery; and UV, umbilical vein. *P≤0.05; **P≤0.01; ***P≤0.001. Images adapted from an original diagram by Luke Itani/SickKids Hospital, Toronto.

Groupwise characteristics are displayed in the Table and Figure 3. Between the false positive and true positive groups, there was no significant difference in the mean gestational age at delivery (38.5 versus 38.1 weeks, *P*=0.223), birth weight (3005 versus 3032 g; *P*=0.874), or head circumference (33.8 versus 33.7 cm; *P*=0.369). Groupwise summaries of all phase-contrast flow data are shown in Figure 4 and Table II in the Data Supplement.

**Table.**
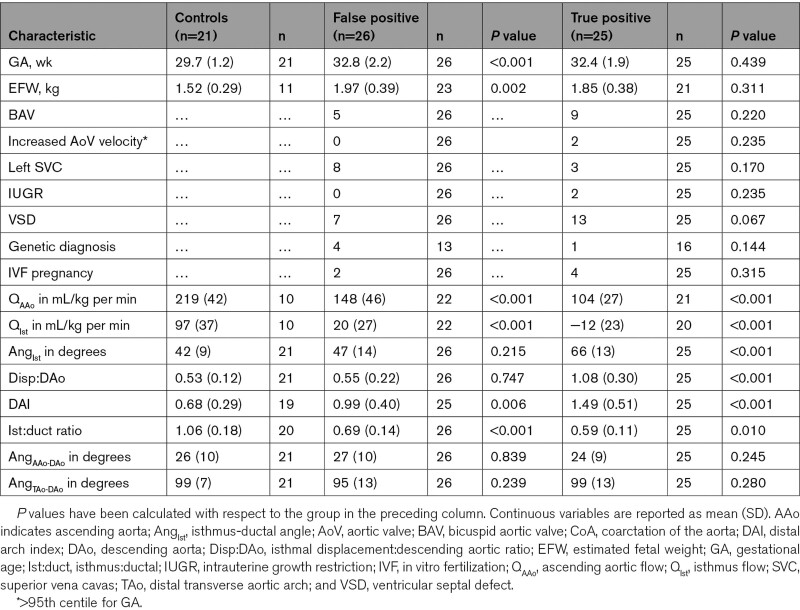
Cohort Characteristics

**Figure 5.**
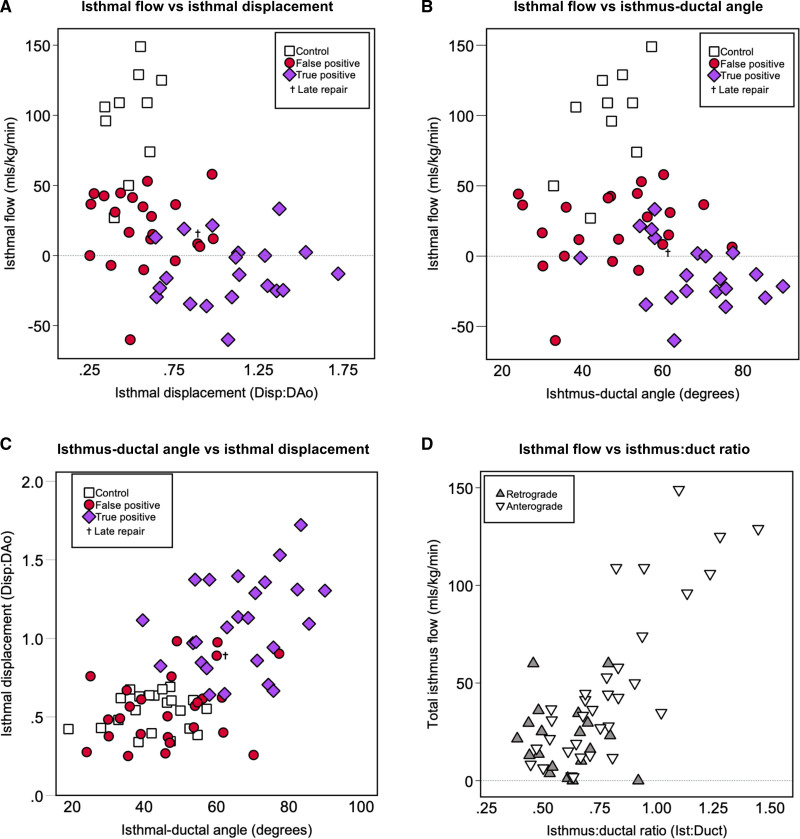
**Scatter plots showing the relationship between vascular flow and anatomy across all cases.** The upper two plots demonstrate the relationship between isthmal flow (Q_Ist_) and both the angle of insertion (**A**) and the proximal displacement (**B**) of the aortic isthmus. The relationship between isthmal angle (Ang_Ist_) and displacement is shown in panel **C**. Total isthmal flow for all cases (anterograde or retrograde) as a function of the isthmus:ductal ratio (IDR) is shown in panel **D**. Disp:DAo indicates isthmal displacement:descending aortic ratio.

Scatter plots showing the relationship between Q_Ist_, Ang_Ist_, and the Disp:DAo ratio are shown in Figure 5A and 5B. There was a negative correlation between Q_Ist_ and both Ang_Ist_ (Spearman correlation coefficient, −0.42; *P*=0.005) and the Disp:DAo ratio (correlation coefficient, −0.2; *P*=0.006). These two measures also showed some correlation with each other (correlation coefficient, 0.61; *P*<0.001; Figure 5C). Finally, there was significant correlation between the isthmus:ductal ratio and absolute isthmal flow (ie, the total flow across the isthmus in any direction; correlation coefficient, 0.57; *P*<0.001; n=42; Figure 5D).

Repeated measurements from the same observer showed excellent agreement with an ICC of 0.95 for all vessel diameters, 0.93 for Ang_Ist_, and 0.95 for the Disp:DAo ratio. Between observers, agreement was excellent for vessel diameters (ICC, 0.92) and good for Disp:DAo (ICC, 0.84) and the distal arch index (ICC, 0.81). There was moderate interobserver agreement for Ang_Ist_ (0.73) and Ang_TAo-DAo_ (0.63). Interobserver agreement was poor for Ang_AAo-DAo_ (0.46).

### Predictive Value of Individual Measures

**Figure 6.**
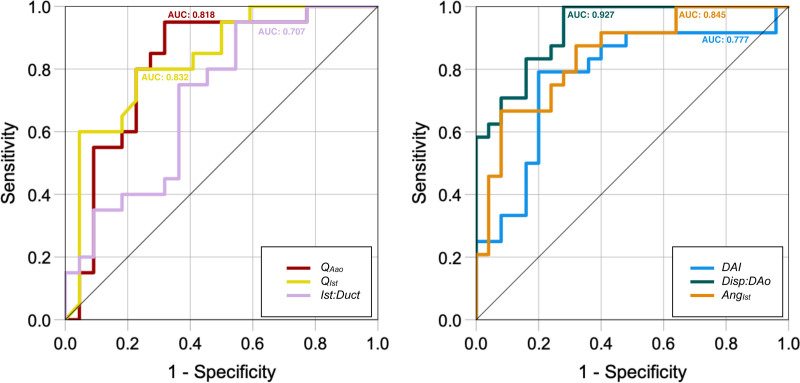
**Receiver operating characteristic curves for the parameters assessed in this study.** Lower values of the factors in the **left** and higher values of those in the **right** were associated with a greater risk of postnatal coarctation. Ang_Ist_ indicates isthmus-ductal angle; DAI, distal arch index; Disp:DAo, isthmal displacement:descending aortic ratio; Q_AAo_, ascending aortic flow; and Q_Ist_, isthmal flow. AUC indicates area under the curve, and Ist:Duct, isthmus ductal ratio.

Results from the univariate regression analysis of the suspected CoA group suggested that a higher risk of postnatal coarctation was associated with reduced ascending aortic flow (*P*=0.003) and Q_Ist_ (*P*=0.002), an increasing Disp:DAo ratio (*P*<0.001), an increased Ang_Ist_ (*P*<0.001), increased distal arch index (*P*=0.002), and a smaller isthmus:ductal ratio (*P*=0.02). A full summary of these results is shown in Table III in the Data Supplement, with ROC curves for each variable shown in Figure 6. We could not demonstrate a significant effect from variation in Ang_TAo-DAo_ (*P*=0.23) or Ang_AAo-DAo_ (*P*=0.24). The same was true when both parameters were analyzed on a per-observer basis.

Multivariate logistic regression revealed Q_AAo_/Q_Ist_ and Disp:DAo to be the only significant predictors of postnatal coarctation in this cohort. A model incorporating Q_AAo_ and Disp:DAo explained 78% of the variance (Nagelkerke R^2^) in outcome and correctly classified 95% of true positive and 91% of false positive cases (overall accuracy, 93%; n=40). A risk assessment table using these two parameters is shown in Table IV in the Data Supplement. A model using Disp:DAo alone explained 72% of the variance and correctly classified 84% of true positive and 83% of false positive cases (overall accuracy, 86%; n=51). The addition of postnatally confirmed bicuspid aortic valve, increased fetal aortic valve Doppler velocity (>95% centile for gestational age), persistent left sided SVC, intrauterine growth restriction, an abnormal genetic result, and in vitro fertilization had no effect on model accuracy.

## Discussion

Predicting severe neonatal CoA before birth is notoriously challenging, with a high burden of false positives.^10^ In this study, we have applied novel 3D anatomic MRI and phase-contrast techniques in fetuses with suspected CoA, in an attempt to more accurately define the fetal phenotype at risk of needing early surgical intervention. To our knowledge, this is the first time fetal cardiac MRI has been applied in this way. Our results show that reduced blood flow though the left heart is associated with important configurational changes at the aortic isthmus in fetal life, which in turn appear to relate closely to the development of critical CoA after birth.

Just under half (25 of 51; 49%) of the cases referred after fetal echocardiography went on to require neonatal surgery in this study, with the remainder initially discharged without intervention following closure of the DA. This false positive rate is broadly in keeping with the accuracy reported in previous studies.^17,23^ However, one striking finding in the current study is that the false positive fetuses were still subnormal in almost every metric compared with the cohort of healthy control patients. Similar findings have been described in previous reports.^7,22,24^ We could not show any significant differences in the prevalence of persistent left sided SVC, bicuspid aortic valve, ventricular septal defects, intrauterine growth restriction, birth weight, or postnatal head circumference in our cohort. To date, only 1 false positive patient has gone on to require late coarctation repair (at 88 days). This is consistent with our previous observations in this patient group, in whom a minority will present with coarctation many weeks after confirmed ductal closure.^6^

The aortic isthmus represents a critical watershed between the left and right ventricular output in fetal life.^25^ Given that SVC flow (≈cerebral blood flow) was consistent across all groups, reduced blood flow in the AAo was, therefore, associated with reduced forward flow at the isthmus, and ultimately, flow reversal as the aortic arch received blood in a retrograde fashion from the DA. Aortic and isthmal flows appeared to vary closely with both angulation (Ang_Ist_) and displacement (Disp:DAo) of the isthmus.^14^ Significantly, this geometric disruption in turn seems to relate closely to the risk of postnatal CoA as the DA closes in postnatal life lending support to the theory proposed by Hutchins 40 years ago.^14^ A prediction model incorporating flow in the AAo and isthmal displacement correctly classified 95% of cases with true coarctation and 91% of the false positive cases.

To our knowledge, there is 1 single echocardiographic study to date, from our own center, which has attempted to generate a predictive model of CoA using midtrimester measurements of the distal transverse aortic arch and the DA at a median gestational age of 23 weeks. An echocardiographic model using these parameters explained 18% of the outcome variance and correctly classified 63% of suspected cases. The highest predicted risk from these measures in the midtrimester was 89%. The current study showed a predictive accuracy >99% but at a more advanced gestation and based on prior suspicion of CoA based on echocardiography, suggesting a potential application to improve diagnostic confidence in selected cases on sequential review. Finally, while the application of MRI only in the third trimester is a potential limitation, the reliability and reproducibility of novel motion-corrected 3D MRI methods compares favorably to fetal echocardiography, which can be hindered by fetal position and sonographic access.^18,26^ More widespread dissemination and development of novel MRI techniques, combined with advances in fetal echocardiography, may help to further improve the diagnosis of CoA in clinical practice.

There are several potential explanations for reduced left ventricular output in fetal life. The association between mitral and aortic valve disease and CoA is well described,^7,8,27–29^ and the presence of a bicuspid aortic valve, with or without appreciable stenosis, has been shown to alter flow dynamics in AAo.^30,31^ Interestingly, in keeping with previous echocardiographic studies,^9^ we observed an increased prevalence of bicuspid aortic valve in both the confirmed CoA and the false positive groups. Second, reduced flow through the left heart may be a consequence of impaired ventricular filling, for example, due to a primary issue with diastolic dysfunction or reduced flow at the foramen ovale.^32,33^ An MRI study in a fetus with borderline left ventricle and postnatal coarctation showed an increase in left-to-right shunting at the foramen ovale with maternal hyperoxygenation (which increases pulmonary venous return to the left atrium), suggesting restrictive ventricular physiology in this specific case.^34^ Conversely, in a study of fetuses with established restriction of flow across the foramen ovale (inferred by an aneurysmal foramen flap), maternal hyperoxygenation led to a clear increase in left ventricular output.^35^ However, only 2 of these 12 fetuses went on to develop CoA in that study. Third, there is a well described evidence of a link between coarctation and a more widespread systemic vasculopathy, potentially responsible for reduced distensibility of the aorta in fetal life and the longer term vascular complications associated with the condition, even after surgical repair.^36,37^ Finally, in some fetuses, it may be the effects of changes in cerebroplacental resistance, such as those seen in intrauterine growth restriction or placental insufficiency, that lead to disrupted flow patterns at the aortic isthmus.^25,38^

In terms of the isthmal size itself, it is logical that increasing flow at the isthmus (in any direction) will lead to a corresponding increase in its cross-sectional diameter, as Rudolph et al^15^ speculated in their seminal animal studies in the early 1970s. It is noteworthy, therefore, that the absolute isthmal flow in our study, irrespective of direction, showed the strongest relationship with the isthmus:ductal ratio, that is, the size of the isthmus may simply be a function of the volume of blood flowing though it, regardless of whether this is retrograde or anterograde. This observation may account for the poor specificity of isthmal size alone in predicting coarctation, both in our cohort and in previous echocardiographic studies.^8,39^

Finally, while there did seem to be a relationship between CoA and an increased distance between the left common carotid and subclavian artery (as measured by the distal arch index), we found this to be poorly specific, in keeping with postnatal echocardiographic studies.^21^ We also included the relationship between the angle between the AAo and the DAo (Ang_AAo-DAo_) and the angle between the transverse arch and the DAo (Ang_TAo-DAo_), both of which have been reported to have high sensitivity in a single retrospective study of 40 subjects by fetal echocardiography.^22^ Neither measure was found to have a significant association with postnatal coarctation in our study. It should be noted, however, that the AAo can be difficult to visualize on the black blood reconstructions used, due to the proximity to adjacent vessels and to the fetal heart; this measure also showed the poorest correlation between observers. Recent innovations in fetal MRI reconstruction methods may improve imaging in this area and allow for more detailed 3D revaluation of the great vessels closer to the heart.^40^

### Future Directions

The findings in this article represent preliminary observational data from novel fetal MRI techniques, combined for the first time in vivo. Exciting expansions of these methods may provide an even more sophisticated means of assessment, including fully spatiotemporally resolved volumes of the beating fetal heart,^41^ allowing for functional and volumetric assessment, and even the possibility of generating fetal 4-dimensional flow datasets from prenatal MRI data.^42^ In concert with advanced echocardiographic techniques, these methods may yet provide unprecedented insights into the fetal origins of a wide range of congenital cardiac and extracardiac pathologies. Finally, the concept of chronic maternal hyperoxygenation as a noninvasive means of inducing flow through left heart in utero remains an intriguing possibility.^43^

### Limitations

The referral cohort in this study was based on the discretion of the referring cardiologist and was not representative of all comers to fetal cardiology. The analysis presented may, therefore, not be generalizable to an unselected population. Similarly, all fetuses in this study were scanned in the third trimester; applicability to earlier gestational ages should not be assumed. MRI findings in this study were analyzed by the binary outcome of surgical intervention at <28 days of age. Longer term outcomes, such as late-presenting CoA, are the subject of ongoing investigation; our current unit policy is that all patients remain under postnatal outpatient follow-up for at least 1 year after delivery. Future prospective studies should focus on recruiting consecutive cases with a larger healthy control group, paired fetal echocardiography, with well defined long-term outcomes for both true positive and false positive cases.

### Conclusions

Reduced left ventricular output is seen in both false positive and true positive cases of fetal CoA. A model incorporating AAo flow and proximal displacement of the aortic isthmus using novel MRI methods in the third trimester is highly predictive of critical neonatal coarctation. Alongside fetal ultrasound, fetal cardiac MRI could be a useful adjunct in predicting the need for early postnatal intervention.

## Sources of Funding

This research was funded in whole, or in part, by the Wellcome Trust (102431). For the purpose of Open Access, the author has applied a CC BY public copyright license to any Author Accepted Manuscript version arising from this submission. In addition, the authors acknowledge funding via the Wellcome Trust Engineering and Physical Sciences Research Council (EPSRC) Centre for Medical Engineering, and the Department of Health via the National Institute for Health Research comprehensive Biomedical Research Centre award to Guy's and St Thomas' National Health Service (NHS) Foundation Trust, King's College London and King's College Hospital NHS Foundation Trust. DL also acknowledges support from the Rothschild Foundation (2020/2017).

## Disclosures

None.

## Supplementary Material


