# Supplementary material for: Analysis of 3-Dimensional Arch Anatomy, Vascular Flow, and Postnatal Outcome in Cases of Suspected Coarctation of the Aorta Using Fetal Cardiac Magnetic Resonance Imaging
Source: Circ Cardiovasc Imaging. 2021 Jun 30;14(7):e012411. doi: 10.1161/CIRCIMAGING.121.012411 (PMC8300852; doi:10.1161/CIRCIMAGING.121.012411)
Supplement: Supplementary file 1 [file hci-14-e012411-s001.pdf]

**Supplementary Table 1:** Individual patient characteristics

| No | GA @ MRI | Maternal Hx | Associated features | Confirmed ECA | IUGR | aGCH | GA @ birth | Outcome | Age @ surgery | QAAo | QIst | AngIst | Disp:DAo | DAI | IDR | AAo-DAo | AAo-Tao |
|---|---|---|---|---|---|---|---|---|---|---|---|---|---|---|---|---|---|
| **1** | **36** | **.** | **MV** | **.** | **No** | **Normal** | **38** | **Confirmed CoA** | **6** | **.** | **.** | **53** | **1.0** | **2.45** | **0.53** | **21** | **122** |
| 2 | 36 | HypoThy | VSD | . | No | . | 38 | False Positive | . | . | . | 39 | 0.4 | 0.78 | 0.78 | 17 | 115 |
| 3 | 35 | . | AnFO | . | No | . | 39 | False Positive | . | 105 | 0 | 36 | 0.3 | 0.45 | 0.92 | 36 | 85 |
| 4 | 35 | . | AnFO | . | No | . | 38 | False Positive | . | . | . | 47 | 0.4 | 0.63 | 0.72 | 45 | 65 |
| 5 | 33 | GDM | . | . | No | . | 37 | False Positive | . | . | . | 47 | 0.3 | 0.78 | 0.84 | 14 | 104 |
| 6 | 30 | HypoThy | VSD, ARSA | . | No | . | 40 | False Positive | . | . | . | 46 | 0.3 | 1.91 | 0.61 | 19 | 91 |
| **7** | **33** | **.** | **BAV, VSD** | **.** | **No** | **Normal** | **38** | **Confirmed CoA** | **15** | **100** | **-22** | **90** | **1.3** | **2.18** | **0.39** | **23** | **94** |
| **8** | **30** | **.** | **.** | **.** | **No** | **Normal** | **38** | **Confirmed CoA** | **3** | **.** | **.** | **82** | **1.3** | **1.22** | **0.71** | **21** | **86** |
| 9 | 36 | . | VSD, MV | VACTERL | No | (VUS) | 33 | False Positive | (88)† | 77 | 8 | 60 | 0.9 | 1.57 | 0.44 | 17 | 89 |
| **10** | **32** | **.** | **BAV, BLSVC** | **BilAtr, AnoGen** | **No** | **22q11** | **37** | **Confirmed CoA** | **26** | **127** | **-14** | **66** | **1.1** | **2.32** | **0.48** | **38** | **96** |
| 11 | 31 | RA | BAV, VSD | . | No | Normal | 38 | False Positive | . | 144 | 17 | 30 | 0.5 | 0.77 | 0.47 | 37 | 91 |
| **12** | **31** | **.** | **.** | **.** | **No** | **.** | **38** | **Confirmed CoA** | **6** | **67** | **-16** | **74** | **0.6** | **0.88** | **0.71** | **18** | **90** |
| **13** | **29** | **.** | **VSD** | **.** | **No** | **.** | **38** | **Confirmed CoA** | **21** | **137** | **-25** | **73** | **1.4** | **2.00** | **0.49** | **17** | **125** |
| **14** | **30** | **.** | **BAV, VSD** | **.** | **No** | **Normal** | **38** | **Confirmed CoA** | **9** | **81** | **-34** | **56** | **0.8** | **0.95** | **0.65** | **15** | **114** |
| 15 | 30 | . | BAV, SLSVC | . | No | . | 38 | False Positive | . | 138 | 35 | 36 | 0.6 | 1.08 | 1.02 | 27 | 96 |
| 16 | 31 | . | . | . | No | Normal | 38 | False Positive | . | 154 | -10 | 56 | 1.4 | 1.08 | 0.67 | 28 | 103 |
| **17** | **30** | **IVF** | **BAV, VSD** | **.** | **No** | **Normal** | **39** | **Confirmed CoA** | **5** | **130** | **33** | **57** | **0.5** | **1.54** | **0.64** | **33** | **103** |
| 18 | 30 | IVF | VSD, PAPVD | . | No | Normal | 40 | False Positive | . | 197 | 36 | 25 | 0.8 | 1.45 | 0.71 | 23 | 81 |
| **19** | **32** | **.** | **VSD, BLSVC, iIVC** | **.** | **No** | **Normal** | **40** | **Confirmed CoA** | **7** | **87** | **-30** | **62** | **0.6** | **1.68** | **0.44** | **36** | **108** |
| **20** | **30** | **.** | **VSD** | **.** | **No** | **.** | **38** | **Confirmed CoA** | **7** | **120** | **21** | **54** | **1.0** | **1.38** | **0.53** | **15** | **90** |
| **21** | **31** | **.** | **BAV, VSD** | **.** | **No** | **Normal** | **39** | **Confirmed CoA** | **9** | **102** | **2** | **78** | **1.5** | **1.28** | **0.61** | **26** | **101** |
| 22 | 31 | . | . | Polydactyly | No | . | 38 | False Positive | . | 116 | -4 | 48 | 0.8 | 0.82 | 0.53 | 17 | 72 |
| 23 | 34 | . | BAV, BLSVC | . | No | . | . | False Positive | . | 199 | 41 | 46 | 0.5 | 1.07 | 0.69 | 27 | 88 |
| 24 | 33 | GDM | . | . | No | T21 | . | False Positive | . | 147 | 12 | 39 | 0.6 | 0.86 | 0.81 | 25 | 89 |
| **25** | **30** | **.** | **BAV, VSD** | **.** | **No** | **.** | **38** | **Confirmed CoA** | **7** | **94** | **-23** | **76** | **0.5** | **1.33** | **0.8** | **24** | **97** |
| **26** | **32** | **GDM** | **BAV, TV** | **.** | **No** | **.** | **38** | **Confirmed CoA** | **9** | **71** | **-30** | **86** | **1.1** | **1.19** | **0.7** | **31** | **95** |
| 27 | 34 | . | ARSA, BLSVC | . | No | Normal | . | False Positive | . | 152 | 37 | 70 | 0.3 | 0.63 | 0.54 | 17 | 92 |
| **28** | **33** | **.** | **BAV** | **.** | **No** | **.** | **39** | **Confirmed CoA** | **3** | **137** | **-25** | **66** | **1.4** | **2.22** | **0.67** | **20** | **110** |
| 29 | 30 | . | . | . | No | . | 40 | False Positive | . | 190 | 45 | 54 | 0.4 | 0.90 | 0.69 | 31 | 85 |

| | | | | | | | | | | | | | | | | | |
|---|---|---|---|---|---|---|---|---|---|---|---|---|---|---|---|---|---|
| 30 | 33 | . | VSD, ARSA, BLSVC | . | No | Normal | 38 | False Positive | . | 139 | 15 | 61 | 0.6 | 0.96 | 0.61 | 34 | 108 |
| **31** | **33** | **.** | **VSD** | **.** | **Yes** | **.** | **36** | **Confirmed CoA** | **26** | **.** | **.** | **54** | **1.4** | **0.57** | **0.66** | **8** | **82** |
| **32** | **36** | **.** | **VSD** | **.** | **No** | **.** | **38** | **Confirmed CoA** | **7** | **124** | **2** | **69** | **1.1** | **1.00** | **0.61** | **11** | **69** |
| **33** | **32** | **IVF** | **VSD, MV** | **.** | **No** | **.** | **40** | **Confirmed CoA** | **10** | **69** | | **71** | **0.9** | **1.77** | **0.55** | **19** | **112** |
| **34** | **34** | **IVF** | **.** | **.** | **No** | **Normal** | **37** | **Confirmed CoA** | **5** | **.** | **.** | **44** | **0.5** | **1.46** | **0.62** | **22** | **110** |
| 35 | 32 | . | pAVSD, BAV, ARSA, BLSVC | Pierre-Robin, SBAbn, GDD | No | Chr 8 Duplication | 37 | False Positive | . | 56 | -7 | 30 | 0.4 | 1.66 | 0.54 | 35 | 110 |
| 36 | 33 | . | . | . | No | . | 39 | False Positive | . | 129 | 6 | 77 | 0.9 | 0.79 | 0.50 | 18 | 79 |
| **37** | **31** | **.** | **.** | **.** | **No** | **Normal** | **37** | **Confirmed CoA** | **6** | **164** | **-1** | **40** | **1.1** | **1.44** | **0.60** | **31** | **84** |
| **38** | **33** | **.** | **ARSA, BLSVC** | **.** | **No** | **Normal** | **35** | **Confirmed CoA** | **3** | **123** | **13** | **58** | **0.6** | **0.59** | **0.71** | **35** | **78** |
| 39 | 30 | . | . | . | No | 15q25.2 del | 38 | False Positive | . | 210 | 44 | 24 | 0.3 | . | 0.79 | 19 | 86 |
| 40 | 38 | IVF | BAV, BLSVC | . | No | . | 39 | False Positive | . | 140 | 28 | 56 | 0.6 | 0.82 | 0.77 | 21 | 98 |
| **41** | **29** | **.** | **VSD** | **Premature 30+5** | **Yes** | **.** | **30** | **Confirmed CoA** | **75††** | **116** | **19** | **57** | **0.8** | **.** | **0.65** | **20** | **89** |
| **42** | **32** | **.** | **.** | **.** | **No** | **Normal** | **38** | **Confirmed CoA** | **7** | **91** | **-36** | **76** | **0.9** | **1.48** | **0.48** | **21** | **93** |
| 43 | 31 | . | . | . | No | Normal | . | False Positive | . | 155 | 31 | 62 | 0.4 | 0.60 | 0.54 | 49 | 90 |
| 44 | 31 | . | iIVC | LAI, polysplenia | No | Normal | 39 | False Positive | . | 268 | 12 | 49 | 1.0 | 1.86 | 0.67 | 21 | 113 |
| 45 | 31 | . | . | CDH, SBAbn, GDD, scoliosis | No | 15q25.2 del | 36 | False Positive | . | . | . | 35 | 0.7 | 0.66 | 0.67 | 49 | 88 |
| 46 | 32 | . | . | Congential HypoThy | No | . | 39 | False Positive | . | 155 | 58 | 60 | 1.0 | 0.63 | 0.83 | 28 | 114 |
| 47 | 32 | . | BLSVC | Laryngomalacia | No | 16p11.2 del | 38 | False Positive | . | 133 | 53 | 55 | 0.6 | 1.00 | 0.78 | 13 | 112 |
| **48** | **31** | **.** | **BAV** | **.** | **No** | **Normal** | **38** | **Confirmed CoA** | **8** | **89** | **0** | **71** | **1.3** | **1.92** | **0.63** | **27** | **102** |
| 49 | 31 | . | BLSVC | . | No | . | 38 | False Positive | . | 114 | -60 | 33 | 0.5 | 0.92 | 0.79 | 32 | 77 |
| **50** | **34** | **.** | **pAVSD** | **.** | **No** | **Normal** | **38** | **Confirmed CoA** | **10** | **73** | **-13** | **83** | **1.7** | **1.33** | **0.44** | **17** | **86** |
| **51** | **34** | **IVF** | **MV** | **.** | **No** | **.** | **39** | **Confirmed CoA** | **5** | **91** | **-60** | **63** | **1.1** | **1.49** | **0.46** | **42** | **103** |

*† Late coarctation repair (outpatient)*
*†† Coarctation confirmed <28 days; surgery delayed due to prematurity*

*GA = gestational age; ECA = extracardiac abnormality; IUGR = intrauterine growth restriction; aCGH = array-based comparitive genomic hybridization; QAAo = ascending aortic flow (mls/kg/min); QIst = isthmus flow (mls/kg/min); AngIst = isthmus:ductal angle (degrees); Disp:DAo= isthmal displacement indexed to the descending aortic diameter; DAI = distal arch index; IDR = isthmus:ductal ratio; AAo = ascending aorta; DAo = descending aorta; TAo = distal transverse aortic arch; MV = mitral valve abnormality; CoA = coarctation of the aorta; HypoThy = hypothyroidism; VSD = ventricular septal defect; NIPT = non-invasive prenatal testing; AnFO = aneurysmal foramen ovale; GDM = gestational diabetes mellitus; ARSA = aberrant right subclavian artery; BAV = bicuspid aortic vavle; VUS = variant of unknown significance; BLSVC = bilateral superior vena cavas; BilAtr = biliary atresia; AnoGen = anogenital anomalies; 22q11 = 22q11 microdeletion syndrome; RA = rhematoid arthritis; SLSVC = single left sided superior vena cava; IVF = in-vitro fertilization; PAPVD = partial anomalous pulmonary venous drainage; iIVC = interrupted inferio vena cava; TV = tricuspid valve abnormality; pAVSD = partial atrioventricular septal defect; SBAbn = structural brain abnormality; GDD = global developmental delay; Chr = chromosome; LAI = left atrial isomerism; CDH = congenital diagphramatic hernia; del = deletion*

Supplementary Table 2: Mean vascular flow rates

| Fetal vessel | Units | Control Group | n | False Positive | n | *p-value* | Confirmed CoA | n | *p-value* |
|---|---|---|---|---|---|---|---|---|---|
| **Ascending aorta (AAo)** | mls/kg/min | 219 (42) | 10 | 148 (46) | 22 | *<0.001* | 104 (27) | 21 | *<0.001* |
| | %CVO | 43 (6) | 10 | 31 (6) | 22 | *<0.001* | 25 (5) | 20 | *0.002* |
| **Pulmonary artery (MPA)** | mls/kg/min | 272 (39) | 10 | 302 (100) | 22 | *0.370* | 311 (82) | 20 | *0.753* |
| | %CVO | 54 (6) | 10 | 66 (6) | 22 | *<0.001* | 72 (5) | 20 | *0.002* |
| **Arterial duct (AD)** | mls/kg/min | 170 (46) | 10 | 201 (39) | 21 | *0.061* | 229 (70) | 18 | *0.123* |
| | %CVO | 34 (12) | 10 | 43 (9) | 21 | *0.026*[*] | 53 (8) | 18 | *0.001* |
| **Pulmonary flow (PBF)** | mls/kg/min | 102 (59) | 10 | 112 (74) | 21 | *0.100* | 78 (48) | 18 | *0.097* |
| | %CVO | 20 (10) | 10 | 22 (9) | 21 | *0.525* | 18 (9) | 18 | *0.149* |
| **Superior vena cava (SVC)** | mls/kg/min | 121 (20) | 10 | 126 (35) | 22 | *0.752* | 118 (26) | 20 | *0.404* |
| | %CVO | 24 (4) | 10 | 27 (6) | 22 | *0.191* | 28 (5) | 19 | *0.536* |
| **Aortic isthmus (Ist)** | mls/kg/min | 97 (37) | 10 | 20 (27) | 22 | *<0.001* | -12 (23) | 20 | *<0.001* |
| | %CVO | 19 (6) | 10 | 4 (6) | 22 | *<0.001* | -3 (6) | 19 | *0.001* |
| **Foramen Ovale (FO)** | mls/kg/min | 155 (79) | 10 | 38 (58) | 21 | *<0.001* | 30 (55) | 18 | *0.632* |
| | %CVO | 24 (11) | 10 | 9 (12) | 21 | *0.002* | 8 (12) | 18 | *0.646* |
| **Combined ventricular output (CVO)** | mls/kg/min | 506 (58) | 10 | 481 (123) | 22 | *0.549* | 429 (99) | 20 | *0.149* |
| | %CVO | 100 | 10 | 100 | 22 | - | 100 | 20 | - |

*Flow values denoted as mean measurements (SD). All p-values calculated with respect to previous category.*

**Supplementary Table 3.** Results from univariate logistic regression for individual measures (suspected CoA cases only)

| **Parameter** | **Unit change** | **Odds Ratio for Postnatal CoA (95% CI)** | ***p-value*** |
|---|---|---|---|
| $Q_{AAo}$ (mls/kg/min) (SD) | -5mls/kg/min | 1.21 (1.06, 1.37) | *0.003* |
| $Q_{Ist}$ (mls/kg/min) (SD) | -5mls/kg/min | 1.29 (1.10, 1.51) | *0.002* |
| $Ang_{Ist}$ (degrees) (SD) | +5 degrees | 1.72 (1.27, 2.32) | *<0.001* |
| ***Disp:DAo*** (SD) | +0.1 | 2.19 (1.44, 3.33) | *<0.001* |
| ***DAI*** (SD) | +0.1 | 1.27 (1.09, 1.48) | *0.002* |
| ***Ist:Duct* Ratio** (SD) | -0.1 | 1.84 (1.12, 3.01) | *0.016* |
| $Ang_{AAo\text{-}DAo}$ **(degrees) (SD)** | +5 degrees | 1.20 (0.89, 1.62) | *0.243* |
| $Ang_{TAo\text{-}DAo}$ **(degrees) (SD)** | +5 degrees | 0.88 (0.71, 1.10) | *0.276* |

*AAo = ascending aorta;* $Ang_{Ist}$ *= isthmus-ductal angle; Disp:DAo = isthmal displacement:descending aortic ratio; DAo = descending aorta; TAo = distal transverse artic arch.*
